# Establishing sharp and homogeneous segments in the hindbrain

**DOI:** 10.12688/f1000research.15391.1

**Published:** 2018-08-13

**Authors:** David G. Wilkinson

**Affiliations:** 1 The Francis Crick Institute, 1 Midland Road, London, NW1 1AT, UK

**Keywords:** Boundary formation, hindbrain, segmentation

## Abstract

Studies of the vertebrate hindbrain have revealed parallel mechanisms that establish sharp segments with a distinct and homogeneous regional identity. Recent work has revealed roles of cell identity regulation and its relationships with cell segregation. At early stages, there is overlapping expression at segment borders of the Egr2 and Hoxb1 transcription factors that specify distinct identities, which is resolved by reciprocal repression. Computer simulations show that this dynamic regulation of cell identity synergises with cell segregation to generate sharp borders. Some intermingling between segments occurs at early stages, and ectopic egr2-expressing cells switch identity to match their new neighbours. This switching is mediated by coupling between egr2 expression and the level of retinoic acid signalling, which acts in a community effect to maintain homogeneous segmental identity. These findings reveal an interplay between cell segregation and the dynamic regulation of cell identity in the formation of sharp patterns in the hindbrain and raise the question of whether similar mechanisms occur in other tissues.

## Introduction

Many tissues are patterned by graded signals which regulate the spatial expression of transcription factors that specify regional identity. The initial expression domains of these transcription factors have fuzzy borders, and the proliferation and movements of cells during tissue morphogenesis can potentially cause intermingling between adjacent regions ^1, 2^. Nevertheless, a precise pattern subsequently forms in which all cells within each region have the same identity, and there is a straight interface at the border of adjacent domains. This raises the question of how sharp patterns of regional domains are formed and maintained. One general mechanism, which has been extensively studied, is the segregation of cells and restriction of intermingling between adjacent regions, regulated by effectors—such as cadherins or Eph receptors and ephrins—whose expression is coupled to regional identity. This article will focus on recent studies of the vertebrate hindbrain that have revealed a crucial role of the dynamic regulation of cell identity in the establishment of sharp and homogeneous regional domains.

## Segments and segregation

The hindbrain is subdivided into segments, termed rhombomeres (r1–r7), which express a distinct set of transcription factors and underlie the patterning of neurons and branchial neural crest cells ^3^. These include Hox proteins, Egr2 (Krox20), MafB, and Vhnf1, which act in a network to specify the formation and anteroposterior (A-P) specification of segments ^4, 5^. The spatial expression of these transcription factors is regulated by fibroblast growth factor (Fgf) and retinoic acid (RA) signalling that act in feedback loops to establish a gradient of RA in the hindbrain ^6^. The borders of segmental gene expression initially are ragged and then sharpen over a period of several hours. Clonal analyses revealed that there is extensive intermingling in the neural epithelium, driven by cell intercalation during cell proliferation and convergent-extension movements ^7, 8^. However, once segment borders can be seen morphologically, cell intermingling between rhombomeres is restricted ^7^. Subsequent work revealed key roles of signalling by Eph receptors and ephrins which have complementary segmental expression in the hindbrain in the establishment of sharp borders ^9– 13^. The mechanisms by which Eph-ephrin signalling drives border sharpening remain unclear but, based on studies in other tissues and in cell culture assays, it likely involves cell segregation driven by contact repulsion or increased cortical tension ^14– 17^. Following sharpening, there is Eph signalling-dependent formation of actin cables at rhombomere boundaries, which mediate increased actomyosin-dependent tension required to maintain straight borders ^18^.

## Mutually exclusive identity

**Figure 1.**
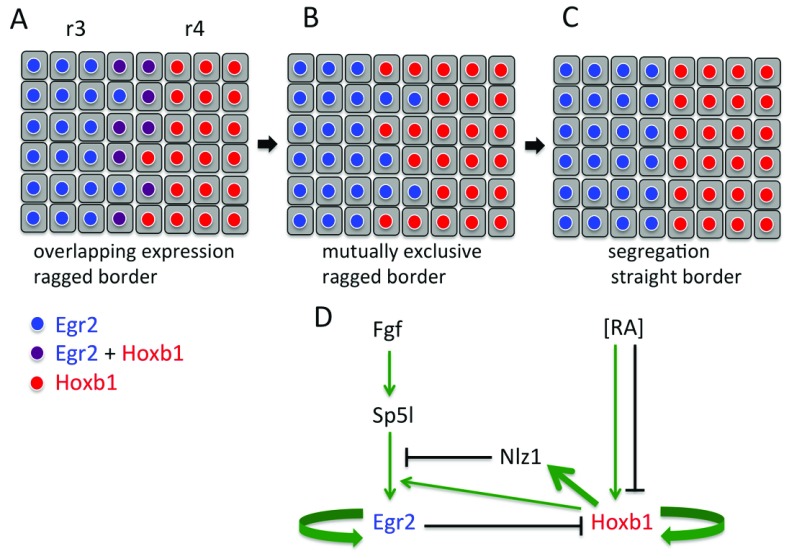
Model of border sharpening by reciprocal repression and cell segregation. ( **A**) At early stages, some cells at the r3/r4 border co-express *egr2* and *hoxb1* because of imprecision in the regulation of segmental gene expression by graded retinoic acid (RA). ( **B**) Reciprocal repression of *egr2* and *hoxb1* leads to mutually exclusive expression, but the border is still ragged. ( **C**) Cell segregation leads to sharpening to form a straight border. The synergistic role of gene regulation and cell segregation is supported by computer simulations. ( **D**) Simplified depiction of the network of gene regulation that upregulates *egr2* in r3/r5 and *hoxb1* in r4 and underlies mutual repression. r, rhombomere.

Evidence that cell identity regulation contributes to the formation of sharp borders has come from studies of *egr2* and *hoxb1*. *egr2* is expressed in and required for the formation of r3 and r5 ^19^, whereas hoxb1 is expressed in r4 and contributes to A-P specification of this segment ^20, 21^. At early stages, some cells at the borders of r4 co-express *hoxb1* and *egr2* ( Figure 1A), and this is likely due to imprecision in the formation and interpretation of graded RA signalling that underlies A-P patterning of the hindbrain ^22^. The overlap in expression is resolved such that cells express one or the other transcription factor ( Figure 1B). Insights into how this transition occurs have come from studies of the regulation of *hoxb1* and *egr2* gene expression ( Figure 1D).

*hoxb1* is initially expressed in a broad domain up to the r3/r4 boundary, which then becomes restricted to r4. The early expression is initiated by RA signalling acting through a conserved 3′ RA response element ^23^. Subsequently, expression in r4 is maintained by an enhancer that binds Hoxb1, Hoxa1, and Hoxb2 as well as Pbx and Meis cofactors and thus involves both auto-regulation and cross-regulation from other hox genes ^5^. Expression of *hoxb1* becomes restricted to r4 through repression in r3 and r5 mediated by a 5′ RA response element and by binding of Egr2 ^5, 24^.

Transcriptional regulation of the *egr2* gene in r3 and r5 also involves elements that initiate and maintain expression ^25– 28^. Consistent with studies of factors required for *egr2* expression ^29– 33^, the initiator elements have combinatorial input from Fgf signalling and Hox transcription factors, including positive input from low levels of Hoxb1 expression ^25, 28^. This drives a pulse of Egr2 protein, which in turn acts on an autoregulatory element that maintains and increases the level of *egr2* expression ^27^. This is counterbalanced by inhibitory loops that limit *egr2* expression to the appropriate level ^34– 36^. Whereas low levels of Hoxb1 promote *egr2* expression, the high-level expression of Hoxb1 in r4 represses *egr2* and acts via the upregulation of Nlz1 ^28^. Remarkably, recent work has found that an enhancer which initiates *egr2* expression in r3 is required for the autoregulatory element to function ^37^. Furthermore, there are long-range physical interactions within the *egr2* regulatory region that likely enable this potentiation of the autoregulatory element by the initiator element ^37^. The cooperation between regulatory elements may help ensure that inappropriate autoregulation does not occur.

*egr2* and *hoxb1* thus act in a bistable switch in which they autoregulate and reciprocally repress each other, such that any cells which initially co-express both transcription factors at segment borders come to express one or the other. Computer simulations suggest that noise in the RA gradient leads to the initial rough borders of gene expression and that fluctuations in *hoxb1* and *egr2* levels enable the transition from co-expression to mutually exclusive expression ^22^. In recent work, a multi-scale model has been developed to simulate both mechanical forces and plasticity of cell identity. These computer simulations have been used to address whether border formation can be accounted for by the generation of mutually exclusive identity or by cell segregation or both ^38^. It was found that cell segregation alone is not able to drive the formation of a sharp border if there is a wide ‘transition zone’ in which there is a mixture of cells with distinct identity (that is, when the border is very fuzzy) ^38^. This is due to the mechanics of cell segregation driven by adhesion or repulsion, which require that cells with the same properties are in contact with each other: isolated cells will segregate to the relevant segment only if they happen to come into contact with it. The regulation of mutually exclusive cell identity facilitated by noise narrows the transition zone but alone is not able to fully sharpen the border ^38^. In contrast, border sharpening does occur in a model in which plasticity in identity and cell segregation are combined ^38^. The dynamic regulation of segmental identity narrows the transition zone and this enables cell segregation to efficiently drive border sharpening. These simulations thus suggest that identity regulation and cell segregation have complementary strengths and act synergistically to sharpen borders ( Figure 1A–C).

## Identity switching

Cell lineage analysis in the chick hindbrain revealed that there is some intermingling between segments at early stages, prior to the formation of morphological boundaries ^7^. Furthermore, single cells transplanted between hindbrain segments in mouse or zebrafish were found to change their identity to match the new location ^39, 40^. Such switching did not occur for groups of transplanted cells, suggesting that cell identity is regulated by community signalling, but the nature of the signals was unknown. Based on these findings, it was proposed that identity switching of cells that intermingle acts in parallel with cell segregation to maintain homogeneous segments ^41, 42^. This idea was challenged by a study which found no intermingling between segments in zebrafish ^18^, but this work used reporters detected during and after border sharpening, when cell segregation mechanisms are in place. A recent study has shown that intermingling between segments does occur in zebrafish and has elucidated the mechanism of cell identity switching ^43^. Since Egr2 is a direct regulator of the *ephA4* gene ^44^, it is likely that intermingling of *egr2*-expressing cells into adjacent segments would occur only during an early time window, when the expression level of EphA4 is not high enough to drive cell segregation. By generating a transgenic reporter line in which expression of a stable fluorescent protein is driven directly from the *egr2* locus, investigators found that some cells do intermingle between r3/r5 and adjacent segments ^43^. These ectopic cells downregulate *egr2* expression and, when present in r4, upregulate *hoxb1* expression ^43^.

**Figure 2.**
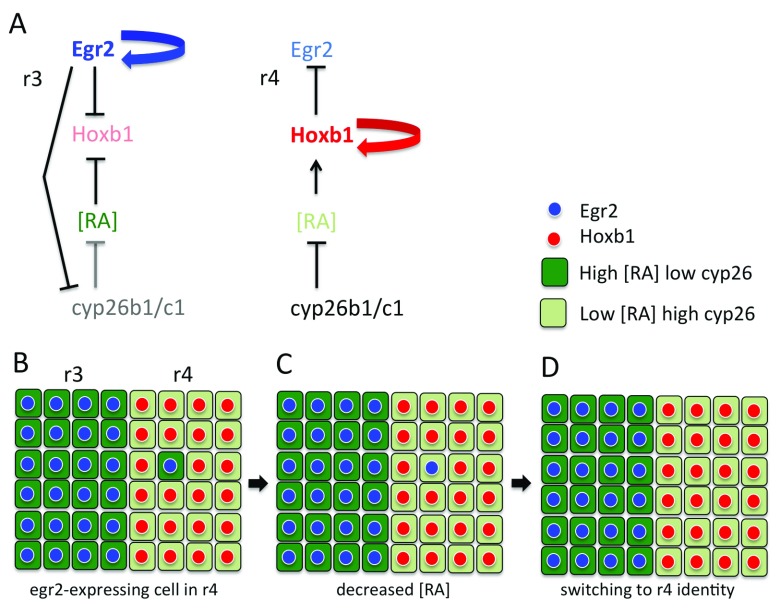
Model of how homogeneous segments are maintained by identity switching. ( **A**) Relationship between *egr2*, *hoxb1*, and retinoic acid (RA) levels regulated by *cyp26b1* and *cyp26c1*. In r3 and r5, high-level *egr2* expression downregulates *cyp26b1* and *cyp26c1* expression. This creates a high-RA environment that suppresses *hoxb1* expression. In r4, there is high *cyp26b1* and *cyp26c1* expression. This creates a low-RA environment that promotes *hoxb1* expression. ( **B**) Owing to coupling between *egr2* expression and *cyp26b1/c1* expression levels, an *egr2*-expressing cell that intermingles into r4 initially has a high-RA level. ( **C**) There is non-autonomous depletion of RA by high *cyp26b1/c1* expression in adjacent cells. ( **D**) This leads to upregulation of *hoxb1* and thus downregulation of *egr2* expression. r, rhombomere.

Previous studies have shown that the degradation of RA by Cyp26 family members has a crucial role in establishing differential levels of RA that underlie A-P patterning in the hindbrain. The *cyp26a1* gene is regulated by the level of RA and Fgf signalling, which leads to expression in an anterior-to-posterior gradient in the early zebrafish hindbrain ^6^. In contrast, *cyp26b1* and *cyp26c1* are not directly regulated by RA signalling and have dynamic segmental expression that progresses from anterior to posterior ^45, 46^. As strong disruption of A-P patterning occurs only after blocking the function of all three family members ^45^, they are thought to have parallel roles in regulating the level of RA. *cyp26b1* and *cyp26c1* are expressed at lower levels in r3 and r5 than in r2, r4, and r6, and it was found that this odd-versus-even difference is regulated downstream of *egr2* ^43^. Since Cyp26 enzymes have a strong cell-autonomous and weak non-autonomous effect on RA levels ^6, 47, 48^, this suggests that r3 or r5 cells that intermingle into adjacent segments move from a high- to a low-RA environment. Furthermore, ectopic expression of *egr2* in even-numbered segments was found to be downregulated following blocking of RA signalling, whereas knockdown of *cyp26b1* and *cyp26c1* leads to a failure to downregulate *egr2* in cells that have intermingled from r3 and r5 ^43^. These findings suggest that *cyp26b1* and *cyp26c1* switch the identity of *egr2*-expressing cells that have intermingled into even-numbered segments by non-autonomously decreasing RA levels ( Figure 2A–D). In r4, the switching involves upregulation of *hoxb1*, which in turn represses *egr2* expression ^43^.

## Relationships between cell segregation and identity

Taken together, these studies have found that cell identity regulation not only acts to refine borders in combination with cell segregation but also maintains the homogeneity of segments despite intermingling that occurs before segregation mechanisms have been upregulated. The latter mechanism involves the coupling of the level of *cyp26b1* and *cyp26c1* expression and RA signalling to segmental identity. Single *egr2*-expressing cells that intermingle switch identity, as they are surrounded by neighbours with a higher level of *cyp26* expression. In contrast, positive feedback between segmental identity and *cyp26* expression potentially maintains RA levels in groups of cells. The coupling thus underlies a community effect that may explain the results of transplantation experiments with single cells or groups of cells ^39, 40^.

Consistent with this model, non-autonomous effects of *egr2* overexpression were found to depend upon cell organisation. In early work, it was shown that forced mosaic expression of *egr2* in the chick hindbrain induced the upregulation of *egr2* in adjacent cells in even-numbered segments ^49^. The recent study in zebrafish found that this non-autonomous induction occurs when cells overexpressing *egr2* are intermingled with r2/r4/r6 cells but not following Eph-mediated segregation of the *egr2*-expressing cells ^43^. This suggests that cell identity regulation depends upon how many neighbours are of the same or different type. The regulation of both *ephA4* and the level of *cyp26b1* and *cyp26c1* expression by *egr2* creates segregated communities with different levels of RA signalling. Cells at a sharp border have a sufficient number of neighbours with the same *cyp26* expression and thus maintain their identity. In contrast, cells with distinct identity that enter the adjacent community are surrounded and switch to match their new neighbours ( Figure 2B–D). It will be interesting to explore whether the sharpening of fuzzy borders ( Figure 1) involves RA signalling dependent upon how many neighbours have the same or different *cyp26* expression level.

These findings raise a number of new questions. It will be important to understand cell identity switching by quantitative modelling of the gene regulatory networks in r3/r5 and r4. How does a change in the level of RA switch the network from one state to the other? Since cells do not switch identity if transplanted between segments at late stages ^40^, autoregulation or other mechanisms (or both) may increasingly lock the network in each segment into one state. A key issue is to measure the segmental level of RA signalling, but, owing to limitations in the sensitivity and noise of the available techniques, this is currently not possible at the spatial resolution of hindbrain segments ^50– 52^. A further important question is how the segmental levels of *cyp26b1* and *cyp26c1* expression are regulated. Since Egr2 usually acts as an activator, the repression of *cyp26b1* and *cyp26c1* expression in r3 and r5 may involve intermediary factors, but Egr2 can in some contexts act as a repressor ^53, 54^. *hox* genes have been found to regulate the expression of components of the RA signalling pathway ^4, 55– 57^, and it will be interesting to test whether this underlies feedback that maintains segmental identity. Finally, a broader issue raised by studies of the hindbrain is whether a similar interplay between dynamic cell identity regulation and cell segregation refines patterning elsewhere, such as other boundaries in the developing brain ^58^, in particular at early stages when there are extensive morphogenetic movements and cell fate is plastic.

## Abbreviations

A-P, anteroposterior; fgf, fibroblast growth factor; r, rhombomere; RA, retinoic acid.

## References

[1] Dahmann C Oates AC Brand M: Boundary formation and maintenance in tissue development. *Nat Rev Genet.* 2011;12(1):43–55. 10.1038/nrg2902 21164524

[2] Batlle E Wilkinson DG: Molecular mechanisms of cell segregation and boundary formation in development and tumorigenesis. *Cold Spring Harb Perspect Biol.* 2012;4(1):a008227. 10.1101/cshperspect.a008227 22214769 PMC3249626

[3] Lumsden A Krumlauf R: Patterning the vertebrate neuraxis. *Science.* 1996;274(5290):1109–15. 10.1126/science.274.5290.1109 8895453

[4] Parker HJ Krumlauf R: Segmental arithmetic: Summing up the *Hox* gene regulatory network for hindbrain development in chordates. *Wiley Interdiscip Rev Dev Biol.* 2017;6(6):e286. 10.1002/wdev.286 28771970

[5] Tümpel S Wiedemann LM Krumlauf R: *Hox* genes and segmentation of the vertebrate hindbrain. *Curr Top Dev Biol.* 2009;88:103–37. 10.1016/S0070-2153(09)88004-6 19651303

[6] White RJ Nie Q Lander AD: Complex regulation of *cyp26a1* creates a robust retinoic acid gradient in the zebrafish embryo. *PLoS Biol.* 2007;5(11):e304. 10.1371/journal.pbio.0050304 18031199 PMC2080651

[7] Fraser S Keynes R Lumsden A: Segmentation in the chick embryo hindbrain is defined by cell lineage restrictions. *Nature.* 1990;344(6265):431–5. 10.1038/344431a0 2320110

[8] Kimmel CB Warga RM Kane DA: Cell cycles and clonal strings during formation of the zebrafish central nervous system. *Development.* 1994;120(2):265–76. 8149908 10.1242/dev.120.2.265

[9] Xu Q Alldus G Holder N: Expression of truncated Sek-1 receptor tyrosine kinase disrupts the segmental restriction of gene expression in the Xenopus and zebrafish hindbrain. *Development.* 1995;121(12):4005–16. 8575301 10.1242/dev.121.12.4005

[10] Xu Q Mellitzer G Robinson V: *In vivo* cell sorting in complementary segmental domains mediated by Eph receptors and ephrins. *Nature.* 1999;399(6733):267–71. 10.1038/20452 10353250

[11] Cooke JE Kemp HA Moens CB: EphA4 is required for cell adhesion and rhombomere-boundary formation in the zebrafish. *Curr Biol.* 2005;15(6):536–42. 10.1016/j.cub.2005.02.019 15797022

[12] Kemp HA Cooke JE Moens CB: EphA4 and EfnB2a maintain rhombomere coherence by independently regulating intercalation of progenitor cells in the zebrafish neural keel. *Dev Biol.* 2009;327(2):313–26. 10.1016/j.ydbio.2008.12.010 19135438 PMC2861865

[13] Sela-Donenfeld D Kayam G Wilkinson DG: Boundary cells regulate a switch in the expression of FGF3 in hindbrain rhombomeres. *BMC Dev Biol.* 2009;9:16. 10.1186/1471-213X-9-16 19232109 PMC2656489

[14] Rohani N Canty L Luu O: EphrinB/EphB signaling controls embryonic germ layer separation by contact-induced cell detachment. *PLoS Biol.* 2011;9(3):e1000597. 10.1371/journal.pbio.1000597 21390298 PMC3046958

[15] Fagotto F Rohani N Touret AS: A molecular base for cell sorting at embryonic boundaries: Contact inhibition of cadherin adhesion by ephrin/ Eph-dependent contractility. *Dev Cell.* 2013;27(1):72–87. 10.1016/j.devcel.2013.09.004 24094740

[16] Taylor HB Khuong A Wu Z: Cell segregation and border sharpening by Eph receptor-ephrin-mediated heterotypic repulsion. *J R Soc Interface.* 2017;14(132): pii: 20170338. 10.1098/rsif.2017.0338 28747399 PMC5550979

[17] Canty L Zarour E Kashkooli L: Sorting at embryonic boundaries requires high heterotypic interfacial tension. *Nat Commun.* 2017;8(1):157. 10.1038/s41467-017-00146-x 28761157 PMC5537356

[18] Calzolari S Terriente J Pujades C: Cell segregation in the vertebrate hindbrain relies on actomyosin cables located at the interhombomeric boundaries. *EMBO J.* 2014;33(7):686–701. 10.1002/embj.201386003 24569501 PMC4000087

[19] Schneider-Maunoury S Topilko P Seitandou T: Disruption of *Krox-20* results in alteration of rhombomeres 3 and 5 in the developing hindbrain. *Cell.* 1993;75(6):1199–214. 10.1016/0092-8674(93)90329-O 7903221

[20] Studer M Lumsden A Ariza-McNaughton L: Altered segmental identity and abnormal migration of motor neurons in mice lacking *Hoxb-1*. *Nature.* 1996;384(6610):630–4. 10.1038/384630a0 8967950

[21] Goddard JM Rossel M Manley NR: Mice with targeted disruption of *Hoxb-1* fail to form the motor nucleus of the VIIth nerve. *Development.* 1996;122(10):3217–28. 8898234 10.1242/dev.122.10.3217

[22] Zhang L Radtke K Zheng L: Noise drives sharpening of gene expression boundaries in the zebrafish hindbrain. *Mol Syst Biol.* 2012;8:613. 10.1038/msb.2012.45 23010996 PMC3472692

[23] Marshall H Studer M Pöpperl H: A conserved retinoic acid response element required for early expression of the homeobox gene *Hoxb-1*. *Nature.* 1994;370(6490):567–71. 10.1038/370567a0 7914354

[24] Studer M Pöpperl H Marshall H: Role of a conserved retinoic acid response element in rhombomere restriction of *Hoxb-1*. *Science.* 1994;265(5179):1728–32. 10.1126/science.7916164 7916164

[25] Wassef MA Chomette D Pouilhe M: Rostral hindbrain patterning involves the direct activation of a *Krox20* transcriptional enhancer by Hox/Pbx and Meis factors. *Development.* 2008;135(20):3369–78. 10.1242/dev.023614 18787068

[26] Chomette D Frain M Cereghini S: *Krox20* hindbrain cis-regulatory landscape: Interplay between multiple long-range initiation and autoregulatory elements. *Development.* 2006;133(7):1253–62. 10.1242/dev.02289 16495311

[27] Bouchoucha YX Reingruber J Labalette C: Dissection of a Krox20 positive feedback loop driving cell fate choices in hindbrain patterning. *Mol Syst Biol.* 2013;9:690. 10.1038/msb.2013.46 24061538 PMC3792346

[28] Labalette C Wassef MA Desmarquet-Trin Dinh C: Molecular dissection of segment formation in the developing hindbrain. *Development.* 2015;142(1):185–95. 10.1242/dev.109652 25516974

[29] Maves L Jackman W Kimmel CB: FGF3 and FGF8 mediate a rhombomere 4 signaling activity in the zebrafish hindbrain. *Development.* 2002;129(16):3825–37. 12135921 10.1242/dev.129.16.3825

[30] Walshe J Maroon H McGonnell IM: Establishment of hindbrain segmental identity requires signaling by FGF3 and FGF8. *Curr Biol.* 2002;12(13):1117–23. 10.1016/S0960-9822(02)00899-0 12121619

[31] Marín F Charnay P: Hindbrain patterning: FGFs regulate *Krox20* and *mafB/kr* expression in the otic/preotic region. *Development.* 2000;127(22):4925–35. 11044406 10.1242/dev.127.22.4925

[32] Weisinger K Wilkinson DG Sela-Donenfeld D: Inhibition of BMPs by follistatin is required for FGF3 expression and segmental patterning of the hindbrain. *Dev Biol.* 2008;324(2):213–25. 10.1016/j.ydbio.2008.09.005 18823972

[33] Weisinger K Kayam G Missulawin-Drillman T: Analysis of expression and function of FGF-MAPK signaling components in the hindbrain reveals a central role for FGF3 in the regulation of *Krox20*, mediated by Pea3. *Dev Biol.* 2010;344(2):881–95. 10.1016/j.ydbio.2010.06.001 20553903

[34] Mechta-Grigoriou F Garel S Charnay P: Nab proteins mediate a negative feedback loop controlling Krox-20 activity in the developing hindbrain. *Development.* 2000;127(1):119–28. 10654606 10.1242/dev.127.1.119

[35] García-Gutiérrez P Juárez-Vicente F Gallardo-Chamizo F: The transcription factor Krox20 is an E3 ligase that sumoylates its Nab coregulators. *EMBO Rep.* 2011;12(10):1018–23. 10.1038/embor.2011.152 21836637 PMC3185338

[36] Kayam G Kohl A Magen Z: A novel role for Pax6 in the segmental organization of the hindbrain. *Development.* 2013;140(10):2190–202. 10.1242/dev.089136 23578930

[37] Thierion E Le Men J Collombet S: *Krox20* hindbrain regulation incorporates multiple modes of cooperation between *cis*-acting elements. *PLoS Genet.* 2017;13(7):e1006903. 10.1371/journal.pgen.1006903 28749941 PMC5549768

[38] Wang Q Holmes WR Sosnik J: Cell Sorting and Noise-Induced Cell Plasticity Coordinate to Sharpen Boundaries between Gene Expression Domains. *PLoS Comput Biol.* 2017;13(1):e1005307. 10.1371/journal.pcbi.1005307 28135279 PMC5279720

[39] Trainor P Krumlauf R: Plasticity in mouse neural crest cells reveals a new patterning role for cranial mesoderm. *Nat Cell Biol.* 2000;2(2):96–102. 10.1038/35000051 10655589

[40] Schilling TF Prince V Ingham PW: Plasticity in zebrafish *hox* expression in the hindbrain and cranial neural crest. *Dev Biol.* 2001;231(1):201–16. 10.1006/dbio.2000.9997 11180963

[41] Cooke JE Moens CB: Boundary formation in the hindbrain: *Eph* only it were simple... *Trends Neurosci.* 2002;25(5):260–7. 10.1016/S0166-2236(02)02134-3 11972963

[42] Pasini A Wilkinson DG: Stabilizing the regionalisation of the developing vertebrate central nervous system. *Bioessays.* 2002;24(5):427–38. 10.1002/bies.10085 12001266

[43] Addison M Xu Q Cayuso J: Cell Identity Switching Regulated by Retinoic Acid Signaling Maintains Homogeneous Segments in the Hindbrain. *Dev Cell.* 2018;45(5):606–620.e3. 10.1016/j.devcel.2018.04.003 29731343 PMC5988564

[44] Theil T Frain M Gilardi-Hebenstreit P: Segmental expression of the EphA4 (Sek-1) receptor tyrosine kinase in the hindbrain is under direct transcriptional control of Krox-20. *Development.* 1998;125(3):443–52. 9425139 10.1242/dev.125.3.443

[45] Hernandez RE Putzke AP Myers JP: Cyp26 enzymes generate the retinoic acid response pattern necessary for hindbrain development. *Development.* 2007;134(1):177–87. 10.1242/dev.02706 17164423 PMC1765950

[46] Sirbu IO Gresh L Barra J: Shifting boundaries of retinoic acid activity control hindbrain segmental gene expression. *Development.* 2005;132(11):2611–22. 10.1242/dev.01845 15872003 PMC2833012

[47] Rydeen A Voisin N D'Aniello E: Excessive feedback of Cyp26a1 promotes cell non-autonomous loss of retinoic acid signaling. *Dev Biol.* 2015;405(1):47–55. 10.1016/j.ydbio.2015.06.008 26116175 PMC4529768

[48] Rydeen AB Waxman JS: Cyp26 enzymes are required to balance the cardiac and vascular lineages within the anterior lateral plate mesoderm. *Development.* 2014;141(8):1638–48. 10.1242/dev.105874 24667328 PMC3978838

[49] Giudicelli F Taillebourg E Charnay P: *Krox-20* patterns the hindbrain through both cell-autonomous and non cell-autonomous mechanisms. *Genes Dev.* 2001;15(5):567–80. 10.1101/gad.189801 11238377 PMC312642

[50] Sosnik J Zheng L Rackauckas CV: Noise modulation in retinoic acid signaling sharpens segmental boundaries of gene expression in the embryonic zebrafish hindbrain. *eLife.* 2016;5:e14034. 10.7554/eLife.14034 27067377 PMC4829421

[51] Shimozono S Iimura T Kitaguchi T: Visualization of an endogenous retinoic acid gradient across embryonic development. *Nature.* 2013;496(7445):363–6. 10.1038/nature12037 23563268

[52] Schilling TF Sosnik J Nie Q: Visualizing retinoic acid morphogen gradients. *Methods Cell Biol.* 2016;133:139–63. 10.1016/bs.mcb.2016.03.003 27263412 PMC4933793

[53] Desmazières A Charnay P Gilardi-Hebenstreit P: Krox20 controls the transcription of its various targets in the developing hindbrain according to multiple modes. *J Biol Chem.* 2009;284(16):10831–40. 10.1074/jbc.M808683200 19218566 PMC2667770

[54] Mager GM Ward RM Srinivasan R: Active gene repression by the Egr2.NAB complex during peripheral nerve myelination. *J Biol Chem.* 2008;283(26):18187–97. 10.1074/jbc.M803330200 18456662 PMC2440619

[55] Choe SK Zhang X Hirsch N: A screen for *hoxb1*-regulated genes identifies *ppp1r14al* as a regulator of the rhombomere 4 Fgf-signaling center. *Dev Biol.* 2011;358(2):356–67. 10.1016/j.ydbio.2011.05.676 21787765 PMC3183228

[56] Rohrschneider MR Elsen GE Prince VE: Zebrafish Hoxb1a regulates multiple downstream genes including *prickle1b*. *Dev Biol.* 2007;309(2):358–72. 10.1016/j.ydbio.2007.06.012 17651720

[57] Makki N Capecchi MR: Identification of novel *Hoxa1* downstream targets regulating hindbrain, neural crest and inner ear development. *Dev Biol.* 2011;357(2):295–304. 10.1016/j.ydbio.2011.06.042 21784065 PMC3176680

[58] Kiecker C Lumsden A: Compartments and their boundaries in vertebrate brain development. *Nat Rev Neurosci.* 2005;6(7):553–64. 10.1038/nrn1702 15959467

